# Novel Hydrophobic Polyvinyl-Alcohol Formaldehyde Sponges: Synthesis, Characterization, Fast and Effective Organic Solvent Uptake from Contaminated Soil Samples

**DOI:** 10.3390/molecules27238429

**Published:** 2022-12-02

**Authors:** Yajvinder Saharan, Joginder Singh, Rohit Goyat, Ahmad Umar, Sheikh Akbar

**Affiliations:** 1 Department of Chemistry, Maharishi Markandeshwar (Deemed to be University), Mullana, Ambala 133207, India; 2 Department of Chemistry, Faculty of Science and Arts and Promising Centre for Sensors and Electronic Devices (PCSED), Najran University, Najran 11001, Saudi Arabia; 3 Department of Materials Science and Engineering, The Ohio State University, Columbus, OH 43210, USA

**Keywords:** hydrophobic, sponges, contaminated soil/water system

## Abstract

In the present research work, PVFTX-100, PVFSDS, and PVFT-80 sponges were prepared using polyvinyl-alcohol (PVA) with surfactants triton X-100/sodium dodecyl sulfate (SDS)/Tween 80, respectively, for the removal of organic solvents from polluted soil/water samples. All three obtained sponges were further made hydrophobic using dodecyltrimethoxysilane (DTMS). The prepared sponges were characterized using different spectroscopic techniques and SEM analysis. The peaks obtained near 1050 cm^−1^ and 790 cm^−1^ were attributed to Si-O-C and alkyl side chain C-H stretching vibration that confirmed the formation of desired sponges. The SEM images showed the random roughness with a number of protrusions on sponge surfaces, which further played an important role in the absorption and retention of organic solvents molecules. The Sears method was chosen to calculate the surface area and pore volume of all the synthesized sponge samples. Among all three prepared sponges, the PVFTX-100 sponge showed a high pore volume and large surface area, with a maximum percentage absorption capacity of 96%, 91%, 89.9%, 85.6%, and 80 for chlorobenzene, toluene, diesel, petrol, and hexane, respectively, after eightcycles. The organic solvent uptake using PVFTX-100, PVFSDS, and PVFT-80 sponges is quite a unique and simple technology, which could be employed at a large scale for contaminated soil/water systems.

## 1. Introduction

The oil/organic solvent spills, due to the rupturing of the pipeline, tanker collision, and industrial spills in the ocean and soil surfaces are hazards to the marine ecosystem, as well as tohuman beings [1,2]. In the last decade or so, several oil spill accidents have been reported. To name a few, around 1092 tons of diesel oil were spilled in Shelby County, Alabama, United States [3], a heavy fuel oil spill ~2500 tons was reported in the Saronic Gulf, Salamis, Greece in 2017 [4], crude oil (~138,000 tons) was spilled in the East China sea in 2018 [5], petroleum oil around 1240 tons was spilled in Walsh County, North Dakota, United States in 2019 [6], and the Colonial Gasoline oil pipeline (38,000 tons) spilled in Huntersville, North Carolina, United States in 2020 [7]. These spills severely disturb the aquatic ecosystem and contaminate the environment [1,2]. Approaches with different mechanisms viz. solidifiers, absorbents, dispersants, booms, and skimmers have been employed for the adsorption of organic solvents from water/soil bodies [8,9,10,11]. With the exception of absorbents, all of these methods have some drawbacks, including low recovery rate, secondary contamination, higher time consumption, and non-biodegradable in nature [12,13,14,15]. These days, the main focus is on absorbent substances, as they effectively trap organic solvents from the polluted spill site [16,17].

Sponges are soft, lightweight, porous absorbents, originally consisting of the fibrous skeleton, having unique characteristics, such as high porosity, high pore volume, good mechanical strength, and excellent biocompatibility. The virtue of the above-maintained sponge properties have been used for the manufacturing of surgical and bandage pads, operative implants, mops, and as an organic solvent adsorbent.

After the innovation of polyester in 1941, it was possible for artificial sponges to be synthesized for the first time. However, eleven years later, in 1952, polyurethane foam was prepared for commercial purposes [18]. Jung and Bhushan observed oleophobic surfaces with their characteristic of self-cleaning as well as antifouling in nature inoil-water emulsions [19]. Zhu et al. (2011) synthesized superhydrophobic polyurethane sponges through a solution immersion process and absorbed various types of oils up to 13 times their weight [20]. Jiang et al. reported a super hydrophilic and underwater superoleophobic PAM hydrogel, which was used for the trapping of water from oil-water systems with high removal capacity as well as a conflict with oil fouling [21]. Zhang et al. prepared a cyclic poly (5-hydroxy-1-cyclooctene) gel, which traps the benzene, anisole, tetrahydrofuran, and dichloromethane up to 199 times their weight [22]. Zhao et al. reported a carbon nanotube sponge with high absorption capacity and swelling rate, which absorbed a broad range of organic solvents from 80 to 180 times of their initial weight [23].

Nguyen et al. demonstrated that graphene-modified sponges through a dip-coating technique have superhydrophobicity as well as high absorption capacity for various kinds of oils [24]. However, the absorption competence significantly decreased after the first absorbing cycle [24]. Liu et al. prepared a capture-coalescence-release model that depends on pH-receptive polymer brushes for the removal of water-dispersed oil droplets [25]. Zhu et al. synthesized a polyurethane sponge by coating polysiloxane, which enhanced the strength in a harsh environment and absorption capacity of oils and organic solvents [26]. However, in this method, used materials were highly expensive, and the process of sponge formation was complicated [26]. Further, Pan et al. prepared a novel hydrophobic polyvinyl alcohol-formaldehyde foams and absorbed organic solvents from the water up to 89.3 g·g^−1^ in a few seconds [27]. Huili Peng et al. prepared a superhydrophobic magnetic cellulose sponge using the co-deposition method and showed the high removal efficiency of oil and reusability [28]. Wang et al. designed polyvinyl-alcohol formaldehyde sponges, with an absorption capacity of oil from 4.0 to 14.0 g/g in different solvents [29]. Armando Encinas et al. reported a hydrophobic luffa sponge functionalized with stearic acid and removed more than 99% of the oil after twelve cycles [30].

The absorbents are of three types: inorganic mineral products, organic natural products, and synthetic polymers. The inorganic mineral products are silica, zeolites, and perlite, but they have low absorption capacity (<2 g·g^−1^) [31,32,33]. The organic natural products viz. wool fiber, rice straw, kapok fiber, wood fiber, corn cob, and milkweed fiber showed high absorption capacity and are unstable in an aqueous medium after oil absorption [34,35]. However, synthetic polymers, such as rubber, polyacrylate, and polyolefin exhibit high absorption capacity (40 g·g^−1^) as compared to inorganic/organic products. However, these showed a very slow absorption rate, and rejuvenation after adsorption is complex [36]. Hence, the development of cost-effective, eco-friendly, and easily applicable sponges to trap the spilled organic solvent efficiently is needed.

Herein, we synthesized the polyvinyl-alcohol formaldehyde sponges using polyvinyl-alcohol (PVA) with surfactants triton X-100/sodium dodecyl sulfate (SDS)/Tween 80 and were further employed for effective removal of organic solvents from polluted soil/water systems.

## 2. Results and Discussion

### 2.1. Characterizations and Properties of Prepared Sponges

**Figure 1:**
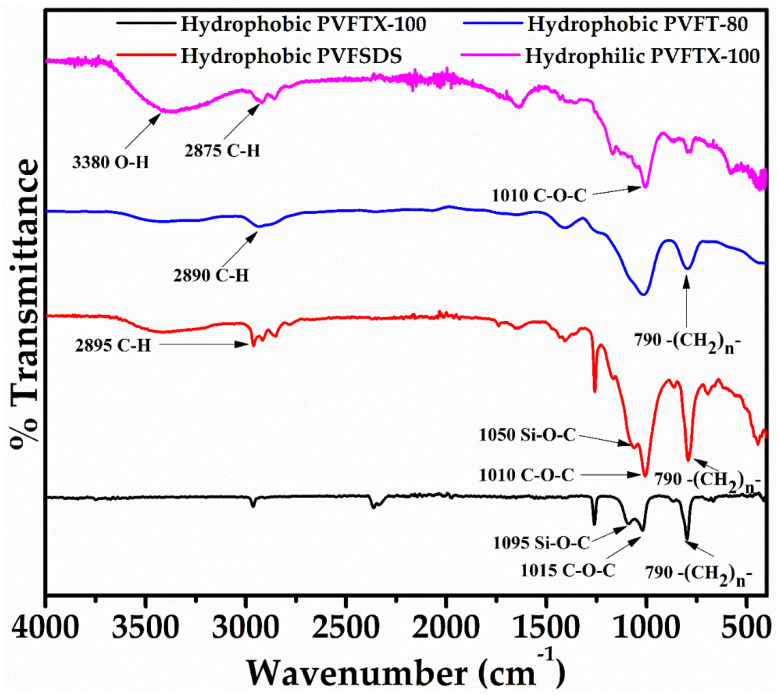
FTIR spectra of hydrophobic PVFTX-100, hydrophobic PVFT-80, hydrophobic PVFSDS, and hydrophilic PVFTX-100 sponges.

Figure 1 displayed the FTIR spectra of all three prepared PVF sponge samples, i.e., hydrophobic PVFTX-100, hydrophobic PVFSDS, hydrophobic PVFT-80, and hydrophilic PVFTX-100. The broad C-OH stretching vibration band around 3380 cm^−1^ was seen in the case of a hydrophilic PVFTX-100 sponge. On the other hand, the absorption intensity was reduced significantly for hydrophobic sponge samples. Further, the peaks observed at 2875 cm^−1^ and 1010 cm^−1^ in all the sponge samples are attributed to the C–H stretching vibration of the alkyl side chain and C-O-C vibration, respectively. The stretching vibrations obtained near 1050 cm^−1^ and 790 cm^−1^ were attributed to Si-O-C and alkyl side chains C-H that finally confirmed the formation of desired sponges [37,38].

**Figure 2:**
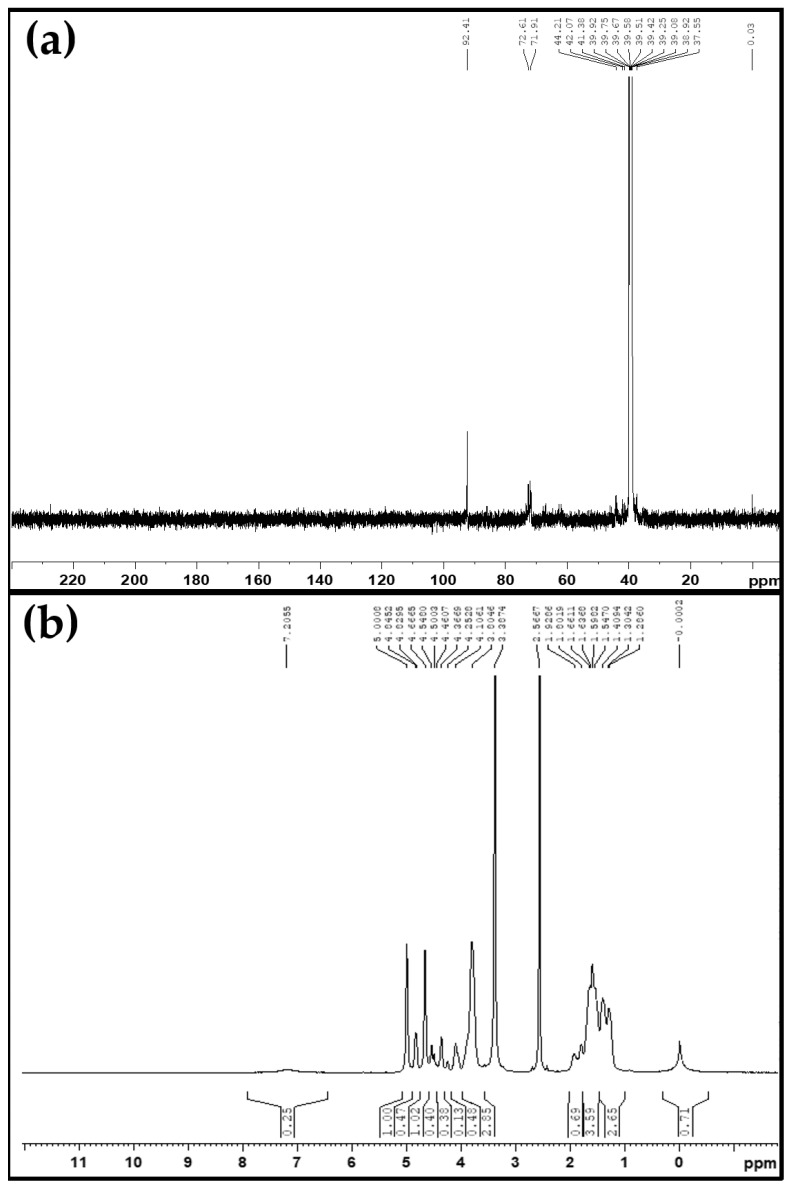
(**a**) ^13^C NMR spectra and (**b**) ^1^H NMR spectra of hydrophobic PVFTX-100 sponge.

Among the three hydrophobic sponge samples, the ^13^C NMR and ^1^H NMR of the hydrophobic PVFTX-100 sponge was studied as it showed the best removal efficiency. The following ^13^C NMR data was obtained from Figure 2a. ^13^C NMR (500 MHz, DMSO), δ37.55 (7-CH_3_), δ 39.58 (6-CH_2_), δ 44.21(5-CH_2_), δ 71.91(4-CH), δ 72.61(3-CH), and δ 92.41(2-CH_2_). A total of six signals were obtained, out of which the signals at δ 37.55 (7-CH_3_) and δ 39.58 (6-CH_2_) were attributed to the 12-carbon side chain (grafting silane chain) contained in the hydrophobic PVFTX-100 sponge, assuring the formation of the hydrophobic sponge [37,39,40]. The following ^1^H NMR data were obtained from Figure 2b. ^1^H NMR (500 MHz, DMSO), δ 1.28 (s, 3H^8^), δ 1.54 (s, 10H^7^), δ 1.92 (s, 12H^6^), δ 3.80 (s, 2H^5^), δ 4.36 (s, 1H^4^), δ 4.46 (s, 1H^3^), δ 4.84 (s, 2H^2^), and δ 7.20 (s, 1H^1^). Total of eight signals were obtained, out of which the three singlets at δ 1.28 (s, 3H^8^), δ 1.54 (s, 10H^7^), and δ 1.92 (s, 12H^6^) were attributed to the structure of −(CH_2_)_n_− (n ≥ 4) and −CH_3_ groups in a grafting silane chain and assured the formation of the required hydrophobic sponge.

**Figure 3:**
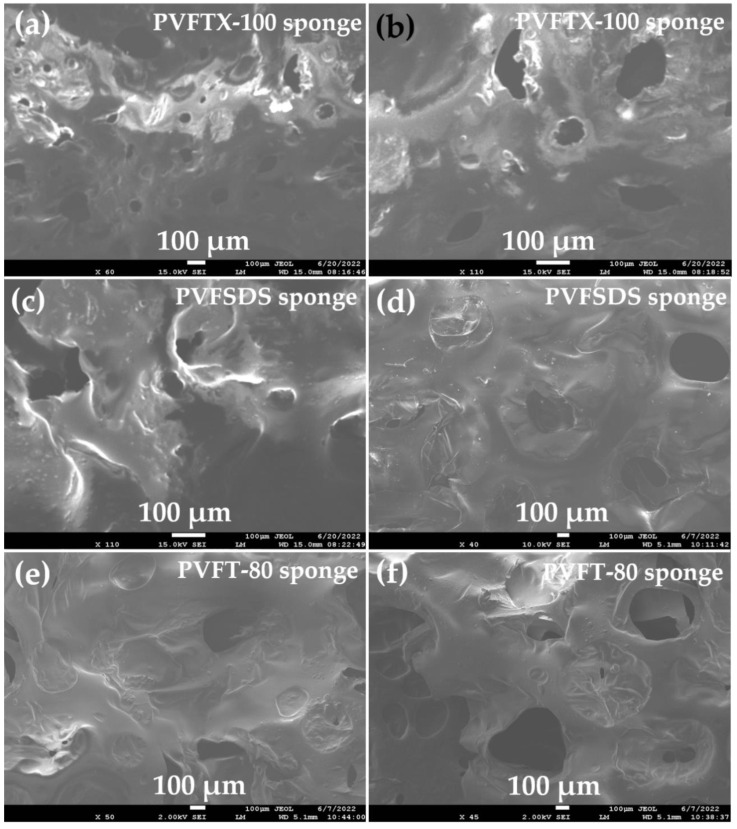
SEM images of (**a**,**b**) PVFTX-100 sponge, (**c**,**d**) PVFSDS sponge, and (**e**,**f**) PVFT-80 sponge.

The SEM images of the synthesized PVFTX-100, PVFSDS, and PVFT-80 sponges as shown in Figure 3. It was observed that the surface of the hydrophobic sponges showed random roughness with many protrusions, which played an important role in the absorption and retention of the solvents. The high roughness nature of the sponge improved the hydrophobicity of the modified sponge, and the size and number of pores in the hydrophobic sponge enabled them more appropriate organic solvents trappers from polluted soil samples [37,41].

As summarized in Table 1, the highest pore volume and surface area were observed in the case of the hydrophobic PVFTX-100 sponge as compared to the hydrophobic PVFSDS and PVFT-80 sponges. The pore volume and surface area of the hydrophobic sponge decreased from 1.21 to 1.05 cm^3^/g and 83.8 to 74.2 m^2^/g, respectively. Further, the hydrophobic PVFTX-100 sponge with elevated surface area (83.8 m^2^/g) and huge pore volume (1.21 cm^3^/g) resulted in an outstanding uptake capacity of 96%, 91%, 89.9%, 85.6%, and 80 for chlorobenzene, toluene, diesel, petrol, and hexane, respectively, as compared to other two sponges.

### 2.2. Visual Observations of Oil Uptake by Hydrophobic PVFTX-100 Sponge

**Figure 4:**
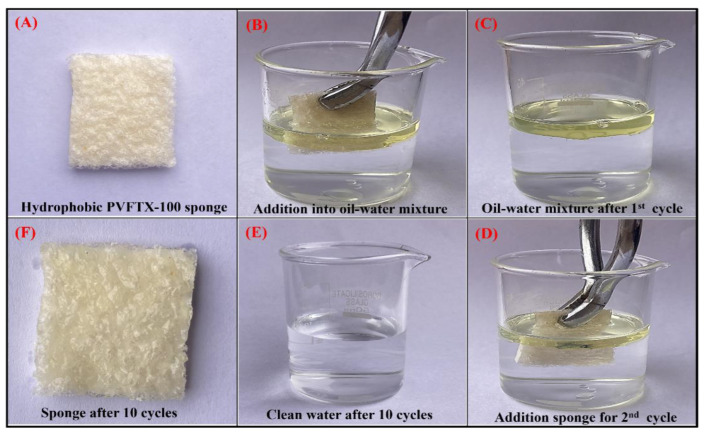
Photographs taken during oil uptake by hydrophobic PVFTX-100 sponge.

The visual observation of the complete trapping of diesel oil using a hydrophobic PVFTX-100 sponge is shown in Figure 4. Initially, a 1.0 g sponge (Picture A) was added to the 40 mL (5 mL of oil and 35 mL of water) oil-water mixture in the ratio of 1:7 (Picture B). After 30 s, the sponge was removed from the mixture, and the weight of the sponge was taken. It was observed that the weight of the hydrophobic sponge increased from 1.0 to 1.61 g. The sponge absorbed 0.61 g of diesel oil from the oil-water mixture (Picture C). After squeezing the sponge, the same procedure was repeated for ten cycles until the complete oil was removed (Picture E). Finally, the clean water and oil-absorbed sponge were obtained as shown in the pictures (E and F), respectively. The oil content absorbed by the hydrophobic sponge was approximately 100% (5 mL), which proved that the hydrophobic modification of the sponge was very effective, and it could accurately separate oil from the oil-water mixture, which provided high application value [42].

### 2.3. Batch Adsorption Studies

**Figure 5:**
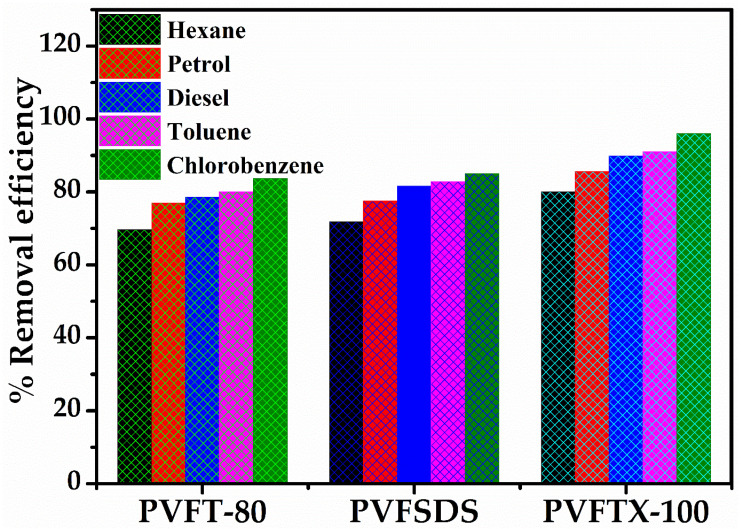
Percentage organic solvents removal efficiency using hydrophobic sponges PVFTX-100, PVFSDS, and PVFT-80.

The results obtained from batch adsorption studies were quite interesting as mentioned in Table 2 and Figure 5. The absorption capacity, basically, depends upon the density of organic solvents. It was observed that the absorption capacity was decreased with decreases in density order from chlorobenzene > toluene > diesel > petrol > hexane, respectively [22,27]. The maximum percentage absorption capacity obtained was 96%, 91%, 89.9%, 85.6%, and 80 for chlorobenzene, toluene, diesel, petrol, and hexane using thePVFTX-100 sponge within 8 cycles. The percentage absorption capacities further decreased when the sponges PVFSDS and PVFT-80 were used for same number of cycles. The obtained results were further supported by the data provided in Table 1. The pore volume and surface area were maximum for PVFTX-100 sponge, showing the highest absorption capacity in comparison to PVFSDS and PVFT-80 sponges. Further, the surface roughness with maximum protrusions shown in SEM images also supports the obtained results.

Further, Table 3 demonstrates the organic solvent uptake capacity comparison of different types of sponges prepared to date. It was noticed that the results of the present studies were quite satisfactory, giving maximum organic solvent uptake with minimum number of cycles as compared to other obtained results from the literature.

## 3. Experimental Details

### 3.1. Materials and Methods

The analytical grade polyvinyl-alcohol (PVA with average molar mass of 13,000–23,000) powder, Triton X-100, sodium dodecyl sulfate (SDS), Tween 80, dodecyltrimethoxysilane (DTMS), formaldehyde, ammonium hydroxide (NH_3_.H_2_O, 25.0%), sulphuric acid (H_2_SO_4_, 98.0%), acetonitrile, toluene, chlorobenzene, and hexane were purchased from Sigma-Aldrich. All the received chemicals were used as such. The diesel and petrol were procured from a local gas station, Mullana, Ambala, India.

### 3.2. Synthesis of Polyvinyl-Alcohol Formaldehyde (PVF) Sponges

(a) Synthesis of PVF Sponge Using Triton X-100 Surfactant

**Figure 6:**
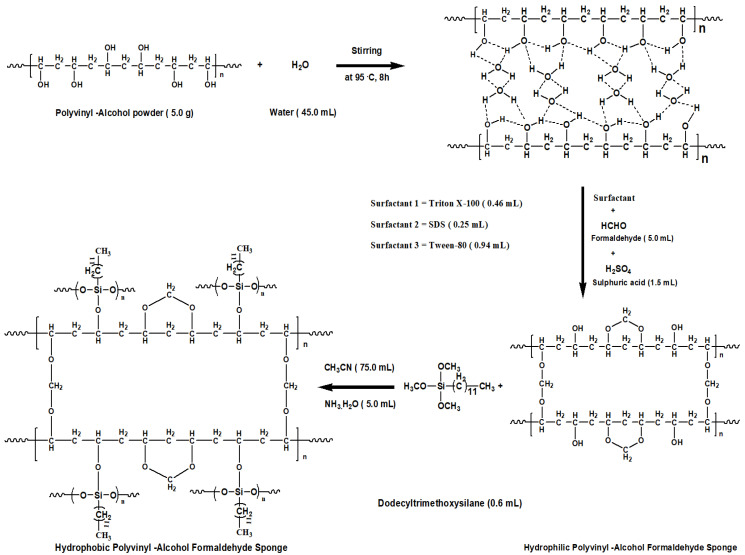
Synthesis and modification of polyvinyl-alcohol formaldehyde (PVF) sponge using different surfactants.

**Figure 7:**
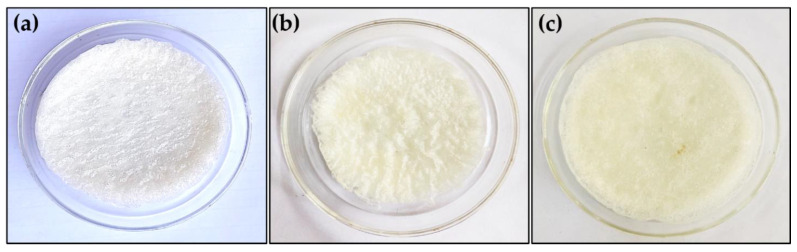
Photographs of the sponges: (**a**) PVFTX-100, (**b**) PVFSDS, and (**c**) PVFT-80.

The PVA powder (5.0 g) and deionized water (45.0 mL) were taken in a 250 mL two-neck round bottom flask (RBF) and were vigorously stirred on a magnetic stirrer at 95 °C for 8 h continuously using an oil bath. On completion of 8 h of stirring, the solution was transferred to a 500 mL beaker and again stirred vigorously at room temperature for 15 min. After that, the surfactant triton X-100 (0.46 mL) and formaldehyde (5.0 mL) were dropped into the above PVA solution. In addition to this 1.5 mL of concentrated sulphuric acid was poured into the reaction system, and finally stirred vigorously so as to obtain the highest foam volume. The obtained foam was poured into a petri dish and oven dried at 70 °C for 16 h. After 16 h oven drying the formed sponge was washed with deionized water 3–4 times to remove any unreacted starting materials as depicted in Figure 6. Finally, the sponge was dried in an oven at 70 °C for 3 h. The obtained sponge was abbreviated as PVFTX-100 (polyvinyl-alcohol formaldehyde sponge based on triton X-100) shown in Figure 7a.

(b) Synthesis of PVF Sponges Using Sodium Dodecyl Sulfate Surfactant

The same procedure as mentioned above was followed, having nearly the same molar concentrations of PVA and SDS surfactant. The obtained sponge was abbreviated as PVFSDS (polyvinyl-alcohol formaldehyde sponge based on sodium dodecyl sulfate) as shown in Figure 7b.

(c) Synthesis of PVF Sponges Using Tween 80 Surfactant

The same procedure as mentioned in Section 3.2 (a) was repeated for the synthesis of the PVFT-80 sponge (polyvinyl-alcohol formaldehyde sponge based on Tween-80) as shown in Figure 7c.

### 3.3. Conversion of Hydrophilic PVF Sponges to Hydrophobic Sponges

The circular obtained PVF sponges (PVFTX-100, PVFSDS & PVFT-80) were cut into square pieces to obtain 1.0 g mass per piece of each sponge. Then, 0.6 mL of DTMS, 75 mL of acetonitrile, and 5.0 mL of NH_3_.H_2_O each were taken in three different 250 mL two-neck round bottom flasks (RBF) and the mixtures were stirred for 10 min at room temperature. After 10 min of stirring, the sponges PVFTX-100, PVFSDS, and PVFT-80 pieces prepared above were added to the reaction mixtures separately and stirred at 50 °C for 6 h. After that, the reaction mixtures were cooled down to room temperature and filtered. The obtained sponges were washed with acetonitrile 4–5 times to remove the unreactive DTMS and were further dried at 60 °C for 12 h [37]. The finally achieved hydrophobic sponges were characterized and used in batch studies for organic solvents uptake from contaminated soil samples.

### 3.4. Characterizations of the Prepared Sponges

The synthesized sponges were examined by different spectroscopic and morphological characterization techniques. The different functional groups of all the sponge samples were examined using Fourier Transform Infrared (FTIR) (Shimadzu 8400S FTIR spectrophotometer from central instrumentation lab MM (DU) Mullana, Ambala, India). The ^13^C NMR spectra was recorded on a Bruker Avance Neo 500 MHz NMR spectrometer (SAIF Lab Chandigarh, Punjab, India) using DMSO as a solvent from to further confirm the chemical structure of hydrophobic sponges [37]. Further, SEM (Scanning electron microscopy: Model JSM6100; Jeol, SAIF Lab Chandigarh, Punjab, India) studied the surface morphologies of the synthesized sponges.

### 3.5. Surface Area Determination of the Prepared Sponges

The surface area of the hydrophobic PVF sponge samples were calculated using the Sear’s method [41,49]. The 1.5 g sample each of the hydrophobic PVFTX-100, hydrophobic PVFSDS, and hydrophobic PVFT-80 sponges were taken individually in three different 500 mL beakers and mixed with 100 mL of water and 30.0 g of NaCl. The resulted mixtures were stirred for 5 min. Further, 0.1 N HCl was added to each mixture making the final volume to 150 mL. Finally, the pH of the mixtures was increased from pH 4.0 to pH 9.0 using 0.1 N NaOH. The volume of 0.1 N NaOH required was noted, and the surface area was calculated using Equation (1).

A = 32V − 25(1)

where A is the calculated surface area of the sponge samples per gram (m^2^/g), and V is the volume of 0.1 N NaOH required to elevate the pH from 4.0 to 9.0.

### 3.6. Determination of Pore (Void) Volume

The 2.0 g each of the hydrophobic PVFTX-100, PVFSDS, and PVFT-80 sponges were immersed in 30.0 mL of deionized water in three different 250 mL RBFs and heated at 100 °C for nearly thirty minutes. The objective of this process was to remove the adsorbed air in the pores of the sponge. After thirty minutes of heating, all three samples were removed from the RBFs and were dried superficially in the folds of simple filter papers by simple pressing. The dried samples were weighed. Finally, the pore volume was calculated using Equations (2) and (3) [49,50].

(2) $$\Delta w={\;w}_{\mathrm{final}}-w_{\mathrm{initial}}$$

(3) $$p_{v}=\frac{\Delta w}{p_{w}}$$

where Δw is the weight (g) difference between initial (w_initial_) and final (w_final_) weights of the sponges, p_v_ is the pore volume (cm^3^/g), and p_w_ is the density of water (g/cm^3^).

### 3.7. Batch Adsorption Studies

The soil samples were prepared according to procedure adopted in our previous article [49,50]. Firstly, 20 g diesel-spiked soil samples (DSS, containing 5.0 mL diesel) were stirred in 100 mL of deionized water for 3 h in a 500.0 mL single-neck RBF. Then, the obtained mixture was filtered, and the filtrate was collected in a separate beaker. Finally, 1.0 g of the PVFTX-100 sponge was immersed to the filtrate solution, and after 30 s, the sponge sample was removed from the beaker, and was squeezed using stainless steel lemon squeezer to remove the maximum oil from the sponge, and calculated the percentage oil uptake capacity [49,50,51] using Equations(4) and (5).

(4) $$\%R=\frac{V_{f}}{V_{i}}\times 100\%$$

(5) $$V_{o}=m_{f}-m_{i}$$

where %R is the percentage oil uptake capacity of the sponge, V_f_ is the volume of oil absorbed by the sponge, V_i_ is the initial oil volume in solution, m_f_ is the final weight of the sponge with absorbed oil, and m_i_ is the initial weight of the sponge before oil adsorption. The sponge was immersed again in the oil water mixture for nearly 8 cycles and the oil was recovered. Following the same methodology, experiments were performed with other organic solvent-spiked soil samples, and absorption capacity was calculated. Exactly the same procedure was followed using PVFSDS and PVFT-80 sponges.

## 4. Conclusions

In conclusion, PVFTX-100, PVFSDS, and PVFT-80 sponges were prepared using polyvinyl-alcohol (PVA) with surfactants triton X-100/sodium dodecyl sulfate (SDS)/Tween 80 for the sorption of organic solvents from contaminated soil/water samples. The surfaces of the three obtained sponges were made hydrophobic in nature using dodecyltrimethoxysilane (DTMS) as the modifier, which further enhances their uptake capacity. The Fourier transform infrared (FTIR) studies of the sponges were carried out and a comparison was done, which authenticated the new Si–O–C bond formation. The SEM images analysis showed the random rough surface with numerous porous, which ultimately increases the organic solvents trapping efficiency. The surface area and pore volume calculated were the highest (83.8 m^2^/g and 1.21 cm^3^/g) for the PVFTX-100 sponge. Further, the batch studies were carried out using the all three sponges, and a maximum percentage absorption capacity of 96%, 91%, 89.9%, 85.6%, and 80 for chlorobenzene, toluene, diesel, petrol, and hexane was obtained using PVFTX-100 sponge in 8 cycles.

## Figures and Tables

**Table 1:** Pore volume and surface area of the hydrophobic PVFTX-100, PVFSDS, and PVFT-80 sponges.

Sponge Samples	Surface Area ± 5 m^2^/g	Pore Volume ± 0.2 cm^3^/g
Hydrophobic PVFTX-100	83.8	1.21
Hydrophobic PVFSDS	77.4	1.09
Hydrophobic PVFT-80	74.2	1.05

**Table 2:** Comparison table of first eight cycles for % removal efficiency of organic solvents using PVFTX-100, PVFSDS, and PVFT-80 sponges.

Sponge Samples	Number of Cycles	Removal Efficiency ± 1 (%)				
		Chlorobenzene	Toluene	Diesel	Petrol	Hexane
PVFTX-100	2	27.0	24.4	24.0	22.7	21.0
	4	53.0	48.2	47.6	44.0	41.4
	6	76.0	71.8	70.5	64.5	60.7
	8	96.0	91.0	89.9	85.6	80.0
PVFSDS	2	23.6	22.5	22.0	21.4	19.0
	4	45.2	43.3	43.4	41.6	36.4
	6	67.0	64.2	64.0	60.6	54.7
	8	85.0	82.8	81.6	77.5	71.8
PVFT-80	2	22.4	22.0	21.8	21.0	18.4
	4	41.8	40.6	40.2	39.0	36.0
	6	63.6	61.3	61.0	59.6	53.3
	8	83.7	80.0	78.6	77.0	69.7

**Table 3:** Comparison of organic solvents uptake capacity by different types of sponges.

Raw Materials	Amount of Sponge Taken (g)	Organic Solvent Uptake Capacity (g/g)	Number of Cycles	References
Poly (5-hydroxy-1-cyclooctene)	1.0	199	Dna	[22]
Ferric nitrate and Ammonium molybdate	1.0	80–180	20	[23]
Polyvinyl alcohol	1.0	89.3	35	[27]
Chitin	1.0	29–58	10	[43]
Cellulose	1.0	65	Dna	[44]
Polyvinyl alcohol	1.0	1.8–7.0	10	[45]
Polyurethane	1.0	25	Dna	[46]
Melamine	1.0	61.0	30	[47]
Polyvinyl alcohol	1.0	4.0	10	[29]
Catechol	1.0	99%	50	[48]
Polyvinyl alcohol	1.0	100%	10	Present work

Dna: Data not available.

## Data Availability

All the data is presented in the manuscript.

## Sample Availability

Samples of the compounds are available with the authors.

## References

[1] Yong J., Chen F., Li M., Yang Q., Fang Y., Huo J., Hou X. (2017). Remarkably simple achievement of super-hydrophobicity, superhydrophilicity, underwater superoleophobicity, underwater superoleophilicity, underwater superaerophobicity, and underwater superaerophilicity on femtosecond laser ablated PDMS surfaces. J. Mater. Chem. A.

[2] Yang Y., Li X., Zheng X., Chen Z., Zhou Q., Chen Y. (2018). 3D-Printed Biomimetic Super Hydrophobic Structure for Microdroplet Manipulation and Oil/Water Separation. Adv. Mater..

[3] Mishra S., Chauhan G., Verma S., Singh U. (2022). The emergence of nanotechnology in mitigating petroleum oil spills. Mar. Pollut. Bull..

[4] Greece Struggles to Clean Up Oil Spill. https://www.dw.com/en/greece-struggles-to-clean-up-relatively-small-oil-spill-as-black-slick-spreads/a-40511068.

[5] (2018). Huge Oil Spills Spreads in East China Sea, Stirring Environmental Fears. New York Times.

[6] (2019). Oil Spill Reported in Walsh County. https://www.kxnet.com/news/local-news/oil-spill-reported-in-walsh-county.

[7] Colonial Pipeline Spill Information—Huntersville, N.C.|NC DEQ. deq.nc.gov.

[8] Chen J., Yang W. (2021). Analysis of Nano-Silicon Dioxide Modified Waste Building Brick Materials in the Application of Adsorption and Removal of Water Pollutants. Sci. Adv. Mater..

[9] Zhou H., Zhang M., Sun S., Wang X. (2021). Synthesis of Coal Gangue-Based Mesoporous X Zeolite with Soft Template and Its Adsorption Methylene Blue. Sci. Adv. Mater..

[10] Chu Y., Pan Q. (2012). Three-Dimensionally Macroporous Fe/C Nanocomposites as Highly Selective Oil-Absorption Materials. ACS Appl. Mater. Interfaces.

[11] Gui X., Zeng Z., Lin Z., Gan Q., Xiang R., Zhu Y., Cao A., Tang Z. (2013). Magnetic and Highly Recyclable Macroporous Carbon Nanotubes for Spilled Oil Sorption and Separation. ACS Appl. Mater. Interfaces.

[12] Zhang M., Wang S., Hu Z., Zhang R., Wang X. (2021). Synthesis and Characterization of Zeolite X Obtained from Coal Gangue for Adsorption of Cu^2+^. Sci. Adv. Mater..

[13] Li G., Xin X., Yu G., Gu Y., Wu Q., Zhou W., Liu C. (2021). Effect of Asphaltene on Threshold Pressure Gradient of Heavy Oil in Porous Media. Sci. Adv. Mater..

[14] Lv M., Wei X., Peng L. (2021). Preparation of Carbon Fiber-Polyacrylamide Composite Hydrogel Based on Surface Electric-Initiated Polymerization. J. Nanoelectron. Optoelectron..

[15] Yan J., Wei Y., Wen Y., Cai H., Xiao J., Wu S., Jin S. (2021). Adsorption and Migration Characteristics of Fluorine in Ash-Sluicing Water in Soils. Sci. Adv. Mater..

[16] Adebajo M.O., Frost R.L., Kloprogge J.T., Carmody O., Kokot S. (2003). Porous Materials for Oil Spill Cleanup: A Review of Synthesis and Absorbing Properties. J. Porous Mater..

[17] Kulawardana E.U., Neckers D.C. (2010). Photoresponsive Oil Sorbers. J. Polym. Sci. Part A Polym. Chem..

[18] Polyurethane Foam Kitchen Sponge. History of Origin—Vortex Power. www.vortex-power.com.

[19] Jung Y.C., Bhushan B. (2009). Wetting Behavior of Water and Oil Droplets in Three-Phase Interfaces for Hydrophobicity/philicity and Oleophobicity/philicity. Langmuir.

[20] Zhu Q., Pan Q., Liu F. (2011). Facile Removal and Collection of Oils from Water Surfaces through Superhydrophobic and Superoleophilic Sponges. J. Phys. Chem. C.

[21] Zhu H., Qiu S., Jiang W., Wu D., Zhang C. (2011). Evaluation of Electrospun Polyvinyl chloride/Polystyrene Fibers as Sorbent Materials for Oil Spill Cleanup. Environ. Sci. Technol..

[22] Zhang K., Lackey M.A., Cui J., Tew G.N. (2011). Gels based on cyclic polymers. J. Am. Chem. Soc..

[23] Zhao M.Q., Huang J.Q., Zhang Q., Luo W.L., Wei F. (2011). Improvement of oil adsorption performance by a sponge-like natural vermiculite-carbon nanotube hybrid. Appl. Clay Sci..

[24] Nguyen D.D., Tai N.-H., Lee S.-B., Kuo W.-S. (2012). Superhydrophobic and superoleophilic properties of graphene-based sponges fabricated using a facile dip coating method. Energy Environ. Sci..

[25] Liu T., Seiffert S., Thiele J., Abate A.R., Weitz D.A., Richteringa W. (2012). Non-coalescence of oppositely charged droplets in pH-sensitive emulsions. Appl. Phys. Sci..

[26] Zhu Q., Chu Y., Wang Z., Chen N., Lin L., Liu F., Pan Q. (2013). Robust superhydrophobic polyurethane sponge as a highly reusable oil-absorption material. J. Mater. Chem. A.

[27] Pan Y., Wang W., Peng C., Shi K., Luo Y., Ji X. (2014). Novel hydrophobic polyvinyl alcohol–formaldehyde foams for organic solvents absorption and effective separation. RSC Adv..

[28] Peng H., Wang H., Wu J., Meng G., Wang Y., Shi Y., Liu Z., Guo X. (2016). Preparation of Superhydrophobic Magnetic Cellulose Sponge for Removing Oil from Water. Ind. Eng. Chem. Res..

[29] Wang B., Yang X., Sha D., Shi K., Xu J., Ji X. (2020). Silane Functionalized Polyvinyl-Alcohol Formaldehyde Sponges on Fast Oil Absorption. Appl. Polym. Mater..

[30] Alvarado-Gómez E., Tapia J.I., Encinas A. (2021). A sustainable hydrophobic luffa sponge for efficient removal of oils water. Sustain. Mater. Technol..

[31] Syed S., Alhazzaa M.I., Asif M. (2011). Treatment of Oily Water using Hydrophobic Nano-silica. Chem. Eng. J..

[32] Yang C., Kaipa U., Mather Q.Z., Wang X., Nesterov V., Venero A.F., Omary M.A. (2011). Fluorous Metal−organic Frameworks with Superior Adsorption and Hydrophobic Properties toward Oil Spill Cleanup and Hydrocarbon Storage. J. Am. Chem. Soc..

[33] Seal S., Sakthivel T., Reid D., Goldstein I., Hench L. (2013). Hydrophobic High Surface Area Zeolites Derived from Fly Ash for Oil Spill Remediation. Environ. Sci. Technol..

[34] Ali N., El-Harbawi M., Jabal A.A., Yin C.-Y. (2012). Characteristics and Oil Sorption Effectiveness of Kapok Fibre, Sugarcane Bagasse and Rice Husks: Oil Removal Suitability Matrix. Environ. Technol..

[35] Husseien M., Amer A., El-Maghraby A., Hamedallah N. (2009). A Comprehensive Characterization of Corn Stalk and Study of Carbonized Corn Stalk in Dye and Gas Oil Sorption. J. Anal. Appl. Pyrolysis.

[36] Yuan X., Chung T.C.M. (2012). Novel Solution to Oil Spill Recovery: Using Thermo degradable Polyolefin Oil Superabsorbent Polymer (Oil−SAP). Energy Fuels.

[37] Saharan Y., Singh J., Goyat R., Umar A., Akbar S., Ibrahim A.A., Baskoutas S. (2022). Novel Supramolecular Organo-Oil Gelators for Fast and Effective Oil Trapping: Mechanism and Applications. J. Hazard. Mater..

[38] Pan Y.X., Liu Z., Wang W.C., Peng C., Shi K., Ji X.L. (2016). Highly Efficient Macroporous Adsorbents for Toxic Metal Ions in Water Systems Based on Polyvinyl Alcohol−Formaldehyde Sponges. J. Mater. Chem. A.

[39] Singh J., Ali A., Kumar R. (2013). Removal of Ni^2+^, Cu^2+^ and Zn^2+^ using different agricultural residues: Kinetic, isotherm modeling and mechanism via chemical blocking. Asian J. Chem..

[40] Pan Y.X., Li B.R., Liu Z., Yang Z.Y., Yang X., Shi K., Li W., Peng C., Wang W.C., Ji X.L. (2018). Superfast and Reversible Thermoresponsive of Poly(N-isopropyl acrylamide) Hydrogels Grafted on Macroporous Poly(vinyl alcohol) Formaldehyde Sponges. ACS Appl. Mater. Interfaces.

[41] Sears G. (1956). Determination of specific surface area of colloidal silica by titration with sodium hydroxide. Anal. Chem..

[42] Singh J., Saharan Y., Goyat R., Vikash K., Algadi H., Akbar S., Baskoutas S., Umar A. (2022). Modified low-temperature synthesis of graphene oxide nanosheets: Enhanced adsorption, antibacterial and antioxidant properties. Environ. Res..

[43] Duan B., Gao H., He M., Zhang L. (2014). Hydrophobic Modification on Surface of Chitin Sponges for Highly Effective Separation of Oil. ACS Appl. Mater. Interfaces.

[44] Lazzari K., Zampieri K., Zanini M., Zattera A., Baldasso C. (2017). Sorption capacity of hydrophobic cellulose aerogels silanized by two different methods. Cellulose.

[45] Wang Q., Li Q., Akram M.Y., Ali S., Nie J., Zhu X. (2018). Decomposable PVA-based super-hydrophobic 3D porous material for effective water/oil separation. Langmuir.

[46] Liang L., Xue Y., Wu X., Donga Y., Meng X. (2019). Self-assembly modification of polyurethane sponge for application in oil/water separation. RSC Adv..

[47] Sun H., Liu Z., Liu K., Gibril M., Kong F., Wang F. (2021). Lignin-based superhydrophobic melamine resin sponges and their application in oil/water separation Author links open overlay panel. Ind. Crops Prod..

[48] Costa P.M., Learmonth D.A., Gomes D.B. (2021). Mussel-Inspired Catechol Functionalisation as a Strategy to Enhance Biomaterial Adhesion: A Systematic Review. Polymers.

[49] Singh J., Saharan Y., Goyat R., Kumar R., Algadi H., Ibrahim A.A., Baskoutas S., Umar A. (2022). Nanoporous and hydrophobic new Chitosan-Silica blend aerogels for enhanced oil adsorption capacity. J. Clean. Prod..

[50] Singh J., Saharan Y., Kumar R., Alothman A.A., Ifseisi A.A., Aljado A.K., Umar A. (2020). Trapping of oil molecules in clathrates: Oil trapping mechanism, soil composition, and thermal studies. J. Mol. Liq..

[51] Singh J., Ali A., Prakash V. (2014). Removal of lead (II) from synthetic and batteries wastewater using agricultural residues in batch/column mode. Int. J. Environ. Sci. Technol..

